# A Mycobacterium ESX-1–Secreted Virulence Factor with Unique Requirements for Export

**DOI:** 10.1371/journal.ppat.0030105

**Published:** 2007-08-03

**Authors:** Bryant McLaughlin, Janet S Chon, Jason A MacGurn, Fredric Carlsson, Terri L Cheng, Jeffery S Cox, Eric J Brown

**Affiliations:** 1 Program in Microbial Pathogenesis and Host Defense, University of California San Francisco, San Francisco, California, United States of America; 2 Department of Microbiology and Immunology, University of California San Francisco, San Francisco, California, United States of America; University of Washington, United States of America

## Abstract

Specialized secretion systems of pathogenic bacteria commonly transport multiple effectors that act in concert to control and exploit the host cell as a replication-permissive niche. Both the Mycobacterium marinum and the Mycobacterium tuberculosis genomes contain an extended region of difference 1 (extRD1) locus that encodes one such pathway, the early secretory antigenic target 6 (ESAT-6) system 1 (ESX-1) secretion apparatus. ESX-1 is required for virulence and for secretion of the proteins ESAT-6, culture filtrate protein 10 (CFP-10), and EspA. Here, we show that both Rv3881c and its M. marinum homolog, Mh3881c, are secreted proteins, and disruption of RD1 in either organism blocks secretion. We have renamed the *Rv3881c/Mh3881c* gene *espB* for ESX-1 substrate protein B. Secretion of M. marinum EspB (EspB_M_) requires both the *Mh3879c* and *Mh3871* genes within RD1, while CFP-10 secretion is not affected by disruption of *Mh3879c*. In contrast, disruption of *Mh3866* or *Mh3867* within the extRD1 locus prevents CFP-10 secretion without effect on EspB_M_. Mutants that fail to secrete only EspB_M_ or only CFP-10 are less attenuated in macrophages than mutants failing to secrete both substrates. EspB_M_ physically interacts with Mh3879c; the M. tuberculosis homolog, EspB_T_, physically interacts with Rv3879c; and mutants of EspB_M_ that fail to bind Mh3879c fail to be secreted. We also found interaction between Rv3879c and Rv3871, a component of the ESX-1 machine, suggesting a mechanism for the secretion of EspB. The results establish EspB as a substrate of ESX-1 that is required for virulence and growth in macrophages and suggests that the contribution of ESX-1 to virulence may arise from the secretion of multiple independent substrates.

## Author Summary

A major mechanism used by pathogenic bacteria for disabling host defenses is secretion of virulence proteins. These effectors are often transported by specialized secretion machines. One such pathway, present in *Mycobacterium* and other Gram-positive genera, is ESX-1 (early secretory antigenic target 6 system 1). Although ESX-1 is required for multiple phenotypes related to the pathogenesis of infection, only three substrates of the secretion machine have been identified to date, and the mechanism by which these substrates are exported is not understood. In our efforts to understand this virulence-related secretion mechanism, we identified a novel substrate and found that its delivery to the ESX-1 machine requires different protein interactions than previously identified substrates. Finally, we present data that the various ESX-1 substrates contribute additively to virulence. These data are incorporated into a model of ESX-1 function.

## Introduction

The cell surface–associated and secreted proteins of pathogenic bacteria promote the uptake of nutrients; facilitate attachment to specific surfaces, cells, or proteins; function in cell wall maintenance and cell division; and offer protection from harsh environmental conditions, including the host immune system. In *Mycobacteria,* there are at least four pathways to secrete proteins—Sec, SecA2, twin-arginine translocase, and the early secretory antigenic target 6 (ESAT-6) system 1 (ESX-1). Much attention has been focused on the ESX-1 pathway because it is required for virulence and for the secretion of ESAT-6 and culture filtrate protein 10 (CFP-10), two major targets of the immune response in infected individuals.

M. tuberculosis ESX-1 is required for virulence in mice, growth in macrophages, and the suppression of macrophage inflammatory and immune responses, including the arrest of phagosome maturation and the reduced expression of IL-12 and TNF-α [1–6]. The homologous M. marinum ESX-1 is required for virulence in zebrafish, growth in macrophages, cytolysis and cytoxicity, and cell-to-cell spread, in addition to ESAT-6 and CFP-10 secretion [7,8]. In zebrafish embryo infections, M. marinum ESX-1 is required for macrophage aggregation and granuloma formation [9]. In *M. smegmatis,* ESX-1, in addition to being required for secretion of ESAT-6 and CFP-10, modulates conjugal DNA transfer [10,11]. In contrast, most strains of *M. ulcerans,* which is closely related genetically to M. marinum and *M. tuberculosis,* but persists in extracellular locations during mammalian infection, lack most of the ESX-1 components as well as orthologs of the genes extending from *Rv3879c* thru *Rv3883c* [12,13]. Although the ESX-1 secretion machinery (Rv3870, Rv3871, and Rv3877) is required for the arrest of phagosome maturation by M. tuberculosis during an infection of macrophages, the known ESX-1 substrates are dispensable [6]. The multiple phenotypes and host responses dictated by the ESX-1 secretory apparatus suggest that there may be additional substrates, components, and regulatory molecules yet to be identified.

Recently, a third ESX-1 substrate, EspA (Rv3616c), was identified [14]. Unlike ESAT-6 and CFP-10, EspA is encoded at a locus distant from the ESX-1 machine, yet this substrate is codependent with both ESAT-6 and CFP-10 for secretion. The mechanism for this interdependence has not been determined, but the interaction between ESAT-6 and CFP-10 in the bacterial cytosol appears to be required for secretion of the heterodimer [15–18]. Presumably, the stable heterodimer is also required for the secretion of EspA.

The M. tuberculosis region of difference 1 (RD1) locus *(Rv3871-Rv3879c)* and the neighboring genes encode the ESX-1 substrates ESAT-6 and CFP-10, as well as core components of the secretion machine [1–3]. These core components include at least two putative SpoIIIE/FtsK ATPase family members (Rv3870 and Rv3871), a proline-rich predicted chromosome-partitioning ATPase (Rv3876), and a putative transporter protein with 12 transmembrane domains (Rv3877). The non-RD1 gene cluster *Rv3616c-Rv3614c* also is required for secretion of the known substrates [14,19]. Additional proteins are likely to be necessary for the assembly of the ESX-1 machinery, because in *M. smegmatis,* genes extending from homologs of *Rv3866* through *Rv3883c* have been shown to be required for ESX-1–mediated secretion [11]; an M. bovis mutant disrupted for the expression of the genes homologous to *Rv3867* through *Rv3869* fails to secrete ESAT-6 and CFP-10 [20]; and in *M. marinum,* the locus required for ESX-1–mediated secretion extends at least from the homolog of *Rv3866 (Mh3866)* to the homolog of *Rv3881c (Mh3881c),* which in this work we rename *espB* (see below) [7].

Although these studies have identified multiple genes required for ESX-1 function, the biochemical interactions necessary for assembly of the secretion machine and for transport of substrates are still not understood. A model for CFP-10 secretion is that the carboxyterminus of the CFP-10 substrate is recognized by Rv3871, which in turn interacts with the integral membrane protein Rv3870 to direct CFP-10 through the secretion pore [15]. The interaction of CFP-10 with Rv3871 is also required for secretion of ESAT-6, suggesting that this is a requisite step in secretion of the ESAT-6/CFP-10 heterodimer by the ESX-1 machine.

Here, we show that Rv3881c and its M. marinum homolog, Mh3881c, are substrates for secretion by ESX-1. For this reason, we have named the gene product of this locus ESX-1 substrate protein B (EspB). In both species, *espB* encodes a gylcine-rich protein with a predicted molecular weight of ∼47 kDa, without any region of apparent similarity to the secretion signal of CFP-10 or other known secretion signals. Although a substrate of ESX-1, we find that the specific genes required for secretion of EspB differ from those required for the secretion of CFP-10. Biochemical investigation demonstrates that EspB forms a complex with Rv3879c and that Rv3879c interacts with Rv3871, the same component of ESX-1 that interacts with the ESAT-6/CFP-10 complex during its secretion. These data support a model that different substrates are delivered to the ESX-1 machine by molecularly distinguishable pathways. Moreover, each of these pathways for ESX-1–mediated secretion contributes to mycobacterial virulence.

## Results

### Both M. tuberculosis and M. marinum EspB Restore Intracellular Growth to the M. marinum Mutant *espB_M_::tn*

**Figure 1:**
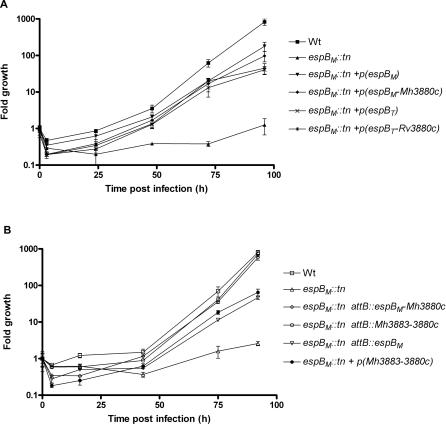
Either EspB_T_ or EspB_M_ Is Sufficient to Restore Growth to the *espB_M_::tn* Mutant in Mouse BMDMs BMDMs were infected at a multiplicity of infection of 1. Colony-forming units (cfu) were determined by lysing infected monolayers and plating lysates at indicated time points. Data were combined for three experiments, with growth indicated as fold increase compared to the initial level of infection for each strain. (A) Wild type (Wt) and *espB_M_::tn* each contain the empty non-integrating plasmid pLYG206; p(*espB_M_*) and p(*espB_T_*) are plasmids for expression of EspB_M_ and EspB_T_, respectively; and p(*espB_M_-Mh3880c*) and p(*espB_T_-Rv3880c*) encode the second gene of the operon as well as *espB* for each species. (B) Wild type (Wt) and *espB_M_::tn* each were transformed with the empty integrating plasmid and the indicated genes were integrated into the *attB* of the *espB_M_::tn* mutant. *espB_M_::tn* + p(*Mh3883c-Mh3880c*) expresses the locus *Mh3883-Mh3880c* on a non-integrating plasmid. Strains differed significantly by one-way ANOVA in (A) after 24 h, 48 h, 72 h, and 96 h and in (B) after 16 h, 43 h, 75 h, and 92 h (*p* < 0.001 for each).

A previous genetic screen for M. marinum mutants that fail to cause hemolysis led to the isolation of eight mutants in the extended RD1 (extRD1) locus, *Mh3866-Mh3881c* [7]. Of the eight mutants, *espB_M_::tn (Mh3881c::tn)* was the most attenuated for virulence to zebrafish, growth in macrophages, and cytotoxicity to J774 cells. Thus, we decided to investigate the *espB_M_*–encoded protein (EspB_M_) and its M. tuberculosis homolog (EspB_T_) in detail. The gene, *espB_M_*, is the first in a two-gene operon. Using quantitative RT-PCR, we found that the mutation disrupts the expression of both genes in the operon (unpublished data). We then sought to determine the genetic requirements for restoration of intracellular growth to the mutant. Introduction of a non-integrating plasmid, expressing either EspB_M_ from the *espB_M_* promoter or EspB_T_ from its native promoter, was sufficient to appreciably restore growth in macrophages to *espB_M_::tn* (Figure 1A). The non-integrating plasmids expressing both EspB_M_ and Mh3880c or both EspB_T_ and Rv3880c were not superior in restoration of intracellular growth. Thus, EspB is necessary and sufficient to appreciably complement *espB_M_::tn,* and the M. tuberculosis homolog functions equally well in *M. marinum,* demonstrating conservation of function.

While the expression of EspB_M_ from a non-integrating plasmid appreciably restored growth in macrophages to *espB_M_::tn,* the complementation was not complete. Among possible explanations are that the transposon insertion exerted a polar effect on the operon upstream, *Rv3883c-Rv3882c,* which also might have a role in intracellular growth, or that a proper stoichiometry between EspB and ESX-1 is required for complete complementation. Therefore, integrating plasmids encoding either *espB_M_* along with the *espB_M_* promoter, *espB_M_-Mh3880c* along with the *espB_M_* promoter, or the entire locus *Mh3883c-Mh3880c* along with the *Mh3883c* promoter, were introduced into *espB_M_::tn.* The locus *Mh3883c-Mh3880c,* along with the *Mh3883c* promoter, was also introduced into *espB_M_::tn* on a non-integrating plasmid. Of these constructs, only the integrating plasmids encoding *espB_M_-Mh3880c* or *Mh3883c-Mh3880c* fully complemented the growth defect of *espB_M_::tn* (Figure 1B). Similarly, *espB_M_* alone appreciably restored a rough colony morphology to the *espB_M_::tn* mutant, but *espB_M_-Mh3880c* or *Mh3883c-Mh3880c* fully restored the rough colony morphology to *espB_M_::tn* (Figure S1). These results suggest that *Mh3880c* can contribute to M. marinum growth in macrophages when it is expressed along with *espB_M_* from the bacterial chromosome. In contrast, *espB_M_* contributes equally well to bacterial virulence whether expressed episomally or on the chromosome, suggesting that its contribution is more independent of its stoichiometry with respect to other virulence components.

### EspB Is a Secreted Protein That Undergoes Carboxyterminal Processing

**Figure 2:**
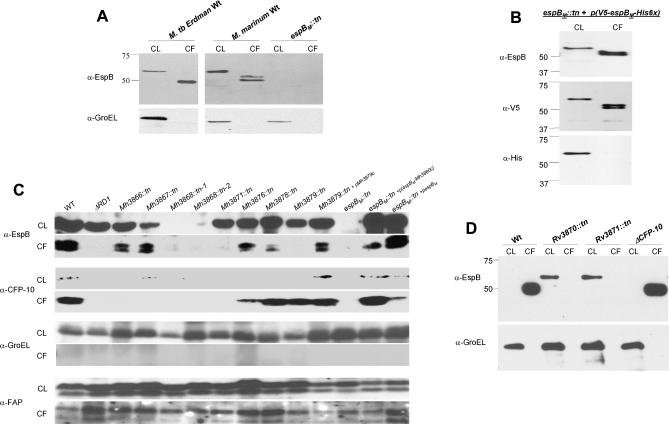
Requirements for EspB Secretion in M. marinum and M. tuberculosis Cell lysates (CL) and culture filtrates (CF) were prepared from the indicated strains as described in Materials and Methods. Proteins were separated by SDS-PAGE, and the indicated proteins were detected by western blot as described in Materials and Methods. (A) 60 μg of total CL and 30 μg of total CF of M. tuberculosis Erdman and each of the M. marinum strains were loaded in each well. (B) 60 μg of total CL and 30 μg of total CF were loaded in each well. (C) Cultures of each strain were grown in 7H9 to an OD of 0.5 and then inoculated into Sauton's medium at an OD of 0.5 and grown for 36 h. Therefore, the samples of each strain are normalized by OD readings. Of the total CL and CF fractions collected for each strain from one experiment, 3% of the CL was loaded in each lane and 15% of the total CF was loaded into each lane. The results shown are representative of the results obtained in four replications of this experiment. (D) 30 μg of total CL and 30 μg of total CF of each M. tuberculosis Erdman strain were loaded in each well.

As a first step toward understanding the role of EspB in virulence and growth in macrophages, we determined its localization in *Mycobacteria* grown in broth culture. The cell lysate and culture filtrate fractions of M. tuberculosis H37Rv, wild-type *M. marinum,* and *M. marinum espB_M_::tn* were probed with a mouse polyclonal antibody raised against a 100 amino acid fragment of EspB_T_ extending from amino acid 234 to 333 (Figure 2A). EspB was detected in both the cell lysate and the culture filtrate fractions of *M. tuberculosis,* as well as in both the cell lysate and the culture filtrate fractions of wild-type M. marinum. EspB was not detected in either fraction of the *espB_M_::tn* culture, verifying the specificity of the antibody. GroEL, a non-secreted bacterial cytoplasmic protein, was found exclusively in the cell lysate, demonstrating that EspB did not appear in the culture filtrate as a result of cell lysis.

The EspB in the cell lysate had an M_r_ of 55 kDa on SDS-PAGE, while the EspB in the culture filtrate of both species ran at a slightly lower molecular weight. A lower molecular weight of EspB in the culture filtrate was also observed in a prior proteomic analysis of M. tuberculosis H37Rv proteins [21], in which EspB in the cell lysate was observed on a 2-D gel as a single spot with an apparent molecular weight of 55.6 kDa, while the EspB in the culture filtrate was observed as two spots with apparent molecular weights of 49.7 kDa and 48.4 kDa. Therefore, EspB might be cleaved either during or after secretion. To test this possibility, a V5 epitope tag was fused to the N-terminus of EspB_M_ and a His6x epitope tag was fused to the C-terminus. The resulting construct, V5-EspB_M_-His6x, was expressed in the *espB_M_::tn* mutant. Like the native protein, V5-tagged EspB was detected in the cell lysate as a single band and as a doublet in the culture filtrate (Figure 2B). In contrast, His-tagged protein was only detected in the cell lysate fraction, suggesting that EspB_M_ in the culture filtrate is C-terminally truncated.

### EspB Secretion Requires a Distinct Set of extRD1-Encoded Genes for Secretion

To assess which ESX-1 genes are required for EspB secretion, its compartmentalization between cell lysate and culture filtrate was determined for several M. marinum ESX-1 mutants (Figure 2C). Although EspB_M_ was found in both the cell lysate and culture filtrate fractions of most mutants, EspB_M_ was not detected in the culture filtrates of *MmΔRD1, Mh3868::tn, Mh3879c::tn,* or *Mh3871::tn*.

The *Mh3868::tn* mutants failed to accumulate protein in the pellet, suggesting that Mh3868 protein could be involved in EspB synthesis or stability. Thus, of the ESX-1 genes tested, only *Mh3879* and *Mh3871* were clearly involved in EspB_M_ secretion. In contrast, none of the mutants secreted ESAT-6 [7], and only *Mh3879c::tn* and *Mh3878c::tn* secreted CFP-10 normally. This difference in secretion requirements for ESAT-6 and CFP-10 in M. marinum has been noted previously [7]. Complementation of *Mh3879::tn* and *espB::tn* restored EspB_M_ secretion. GroEL was absent from culture filtrates of all strains, and secretion of the fibronectin attachment protein (FAP), a protein secreted in a Sec-dependent manner [22], was not disturbed in any of the extRD1 mutants. Thus, the product of the *espB_M_* gene is a secreted protein that requires Mh3871, a core component of the ESX-1 secretion machine, for export; we have therefore named it ESX-1 substrate protein B (EspB). However, EspB, ESAT-6, and CFP-10 differ with respect to the extRD1 genes required for their secretion. EspB_M_ secretion depends on Mh3879c, but is independent of Mh3866 and Mh3867, while CFP-10 shows the inverse pattern.

To demonstrate the importance of the ESX-1 machine in EspB secretion in another strain of *M. marinum,* we examined the 1218R strain and an isogenic mutant in which the *Mh3871* gene had been disrupted. The M strain, used for the previous experiments, is a human isolate, whereas 1218R was originally isolated from an infected fish. Wild-type 1218R secreted EspB, but the *Mh3871* mutant did not (Figure S2), confirming the importance of ESX-1 in the secretion of this protein by M. marinum. Complementation of the mutant with either the M. marinum or M. tuberculosis homolog of *Mh3871* restored secretion of EspB_M_ to this mutant, suggesting parallel functions for the genes in the two species.

To test directly whether ESX-1 was required for EspB secretion by *M. tuberculosis,* we examined culture filtrates from M. tuberculosis Erdman and the isogenic mutants *Rv3870::tn, Rv3871::tn,* and *ΔCFP-10* (Figure 2D). Secretion of EspB_T_ by wild-type M. tuberculosis was abrogated in the *Rv3870* and *Rv3871* mutants, but not in the *ΔCFP-10* mutant. Thus, EspB is a secreted protein in both M. marinum and *M. tuberculosis,* and its secretion requires core ESX-1 components in both species of *Mycobacteria*. Importantly, EspB is the first ESX-1 substrate in M. tuberculosis whose secretion is not disrupted in the *ΔCFP-10* mutant.

### *MmΔRD1, espB_M_::tn,* and *Mh3871::tn* Are More Attenuated for Growth in Macrophages than the Other M. marinum extRD1 Mutants

**Figure 3:**
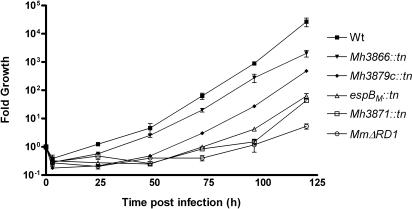
Growth of M. marinum Secretion Mutants in BMDMs BMDMs were infected with M. marinum strains as described in Materials and Methods at a multiplicity of infection of 1, and growth of bacteria was monitored over time as in Figure 1. Data are summarized from three independent experiments. Strains differed significantly by one-way ANOVA after 24 h, 48 h, 72 h, 96 h, and 120 h (*p* < 0.001 for each).

Of the ten M. marinum extRD1 mutants we examined, *MmΔRD1, espB_M_::tn,* and *Mh3871::tn* were disrupted for the secretion of all three substrates: ESAT-6, CFP-10, and EspB_M_. In contrast, the *Mh3879::tn* mutant was disrupted only for the secretion of ESAT-6 and EspB_M_, while the *Mh3866::tn* and *Mh3867::tn* mutants were disrupted only for ESAT-6 and CFP-10 secretion. To assess the importance of the multiple ESX-1 substrates for growth in macrophages, we infected murine bone marrow–derived macrophages (BMDMs) with wild-type *M. marinum,* with strains lacking one secreted effector, or with strains lacking secretion of all the known ESX-1 substrates. As shown in Figure 3, *MmΔRD1, espB_M_::tn,* and *Mh3871::tn,* which fail to secrete all substrates, are more attenuated for growth in macrophages than *Mh3866::tn,* which still secretes EspB_M_, or *Mh3879c::tn,* which still secretes CFP-10. Therefore, we conclude that the various substrates of ESX-1 each contribute to virulence.

### EspB Physically Interacts with the Rv3879c

**Figure 4:**
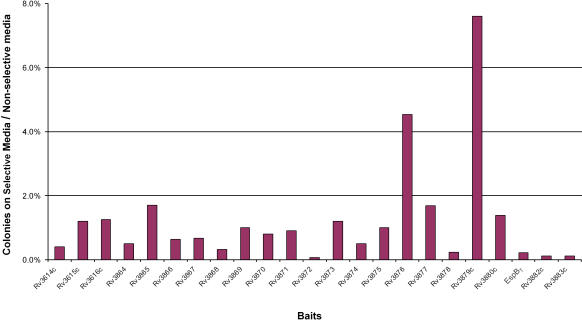
Bacterial Two-Hybrid Analysis of Interaction of EspB_T_ with Proteins of extRD1 The target plasmid containing EspB_T_ fused to the RNA polymerase alpha subunit was co-transformed with each of the bait plasmids containing the indicated extRD1 proteins fused to the lambda repressor into the reporter validation strain. Shown is the ratio of growth of the co-transformants on selective (+5 mM 3AT) versus non-selective plates. The experiment depicted is representative of three independent determinations.

To learn more about the involvement of ESX-1 in EspB secretion, we tested whether EspB would interact with other ESX-1 genes by bacterial two-hybrid analysis (Figure 4). An advantage of the bacterial two-hybrid system is that it can allow detection of interactions of membrane-bound proteins [23]. In this assay, potential protein–protein interactions are assessed by determining the ratio of colonies that grow on selective medium to the number grown on non-selective medium. For each of the bait plasmids, co-transformation with an empty target resulted in a ratio of colonies on selective to non-selective medium of less than 0.1%, as did co-transformation of the EspB_T_ target with an empty bait. In contrast, the Rv3879c bait and EspB_T_ target resulted in a ratio of 7.6%, an increase of more than 75-fold. An Rv3876 bait also showed interaction above background with EspB_T_, but since the *M. marinum Mh3876::tn* mutant showed significant EspB_M_ secretion (Figure 2C), any interaction between Rv3876 and EspB_T_ is not likely to be required for EspB secretion and thus was not pursued.

**Figure 5:**
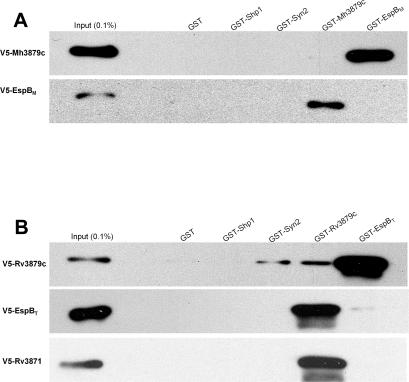
GST Pulldown Analysis of EspB and Rv3879 Interactions (A) Agarose beads with immobilized GST, GST-SHP1, GST-syntaxin2, GST-Mh3879c, and GST-EspB_M_ were incubated with lysates of E. coli that express V5-Mh3879c and V5-EspB_M_. (B) Agarose beads with immobilized GST, GST-SHP1, GST-syntaxin2, GST-Rv3879c, or GST-EspB_T_ were incubated with lysates of E. coli that express V5-Rv3871, V5-Rv3879c, and V5-EspB_T_. Proteins from cell lysates retained on the beads after washing were separated by SDS-PAGE and detected by western blotting with an antibody against V5. To the right of each set of pulldowns, 0.1% of the input E. coli lysate was analyzed. EspB_M_ physically interacts with Mh3879c, EspB_T_ physically interacts with Rv3879c, and Rv3879c also interacts with Rv3871.

To test for an analogous interaction between EspB_M_ and Mh3879c and to confirm the potential interaction between EspB_T_ and Rv3879c suggested by the two-hybrid assay, we performed in vitro pull-down assays. All of the proteins used were expressed in Escherichia coli as GST- or V5-epitope-tagged fusions. Controls for nonspecific interactions included GST alone, as well as GST-syntaxin2, and GST-Shp1. As shown in Figure 5A, GST-tagged EspB_M_, but none of the GST controls, bound specifically to V5-tagged Mh3879c. In the reciprocal experiment, GST-tagged Mh3879c bound specifically to V5-EspB_M_. Similarly, as shown in Figure 5B, GST-tagged EspB_T_ bound specifically to V5-tagged Rv3879c, and GST-tagged Rv3879c bound specifically to V5-tagged EspB_T_. These data demonstrate that recombinant EspB_T_ and Rv3879c, as well as their M. marinum homologs, interact in vitro.

Since *Rv3871* mutants in both M. tuberculosis and M. marinum fail to secrete EspB, we used GST pulldowns to test whether Rv3871 interacts with either EspB_T_ or Rv3879c. GST-tagged Rv3879c bound to V5-tagged Rv3871, whereas the GST controls and GST-EspB_T_ did not bind to Rv3871. This suggests that Rv3879c may facilitate EspB_T_ secretion through an interaction with Rv3871.

### The Carboxyterminus of EspB Is Dispensable for Interaction with Mh3879c and for Secretion

**Figure 6:**
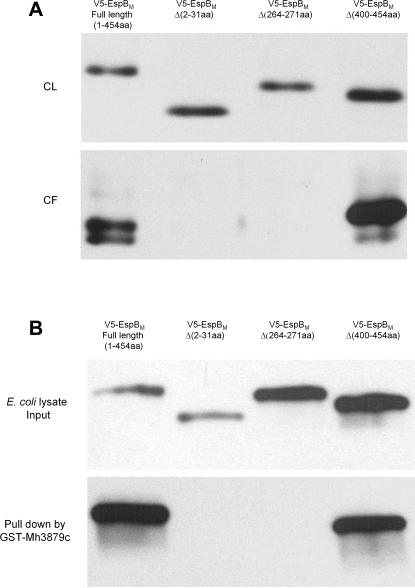
Deletion Analysis of EspB_M_ Secretion and Interaction with Mh3879c (A) The *M. marinum espB_M_::tn* mutant was transformed with a non-integrating plasmid expressing N-terminally V5-tagged EspB_M_ full length, EspB_M_ Δ(2–31), EspB_M_ Δ(264–271), or EspB_M_ Δ(400–454). Cell lysates (CL) and culture filtrates (CF) were prepared from the indicated strains as described in Materials and Methods. The samples of each strain are normalized by OD readings. Of the total CL and CF fractions collected for each strain from one experiment, 3% of the CL was loaded in each lane and 15% of the total CF was loaded into each lane, separated by SDS-PAGE and detected by western blotting with an antibody against V5. (B) Agarose beads with immobilized GST-Mh3879c were incubated with E. coli lysates expressing V5-tagged EspB_M_ full length, EspB_M_ Δ(2–31), EspB_M_ Δ(264–271), or EspB_M_ Δ(400–454). The input lysate (0.1%) and the material from the cell lysates that bound to the beads was run on SDS-PAGE and detected by western blotting with an antibody against V5.

To identify whether EspB, like CFP-10, requires its carboxyterminus for secretion, we constructed a series of EspB_M_ deletion mutants with N-terminal V5 tags and expressed them in the *espB_M_::tn* mutant strain using the *espB_M_* promoter. As shown in Figure 6A, V5-tagged full-length EspB_M_ was secreted. This N-terminally tagged protein, like native EspB_M_, underwent C-terminal truncation either during or after secretion. EspB_M_ deletion mutant constructs Δ(2–31), Δ(264–271), and Δ(400–454) were stably expressed in *M. marinum,* but only EspB_M_ Δ(400–454) accumulated in the culture filtrate. The secreted EspB_M_ Δ(400–454) had a higher apparent molecular weight than the secreted full-length EspB_M_, presumably because deletion of the C-terminal 55 amino acids inhibits some of the carboxyterminal proteolytic processing. This result demonstrates that the C-terminus of EspB_M_ is dispensable for secretion, but N-terminal and internal amino acids are required. Next, we tested how these EspB_M_ mutants interacted with Mh3879c. Lysates of E. coli that express V5-tagged EspB_M_ mutants were incubated with GST-Mh3879c. While full-length EspB_M_ and EspB_M_ Δ(400–454) bound to GST-Mh3879c, the stably expressed but non-secreted EspB_M_ Δ(2–31) and EspB_M_ Δ(264–271) constructs did not bind to GST-Mh3879c (Figure 6B). These data support a model in which EspB interacts with Rv3879c, which in turn interacts with Rv3871, to facilitate the secretion of EspB.

### Mh3879c Is Not Secreted

Because CFP-10 and ESAT-6 are secreted as a heterodimer, we assessed whether Mh3879c and EspB might be secreted similarly. The fusion constructs V5-Mh3879c, Mh3879c-His6x, and V5-Mh3879c-His6x were expressed from the endogenous *Mh3879c* promoter on non-integrating plasmids in both wild-type M. marinum and in the *Mh3879c::tn* mutant. Introduction of V5-Mh3879c fully complemented the EspB_M_ secretion defect of the *Mh3879c::tn* mutant, but Mh3879c-His6x and V5-Mh3879c-His6x failed to complement the secretion defect (Figure S3A). In wild-type *M. marinum,* V5-Mh3879c and V5-EspB_M_ were expressed at nearly identical levels in the cell lysate, but only V5-EspB_M_ was detected in the culture filtrate (Figure S3A). To determine whether failure of secretion reflected inefficient competition of V5-tagged protein with native protein, the V5-EspB_M_ secretion was also analyzed in the *Mh3879::tn* mutant. In this strain as well, V5-Mh3879c was found only in the cell lysate. Thus, V5-tagged Mh3879c, while fully competent to mediate EspB secretion, was not itself secreted, suggesting that Mh3879c and EspB are not secreted as a heterodimer. The C-terminally His6x-tagged Mh3879c, which did not restore EspB secretion to the *Mh3879::tn* mutant, also was detected only in the cell lysate. Since Mh3879c-His6x failed to complement the EspB_M_ secretion defect of the *Mh3879c::tn* mutant, we hypothesized that the carboxyterminus of Rv3879c might be required for interaction with EspB. To test this hypothesis, lysates of E. coli that express V5-tagged Rv3879 mutants were incubated with GST alone, GST-Rv3871, or GST-EspB_T_. While full-length Rv3879 and Rv3879 Δ(1–166) bound to GST-EspB_T_, Rv3879 Δ(564–729) failed to bind to GST-EspB_T_ (Figure S3B). None of the constructs bound to GST alone, and all three constructs bound to GST-Rv3871. Thus, the carboxyterminal 166 amino acids of Rv3879 are required for EspB secretion, but not for interaction with the ESX-1 machine.

## Discussion

**Figure 7:**
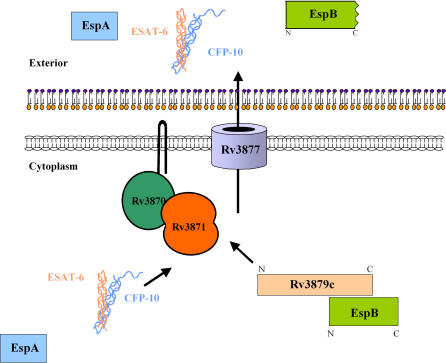
Model for EspB Secretion Depicted are the core ESX-1 components Rv3870, Rv3871, and Rv3877, as well as the ESAT-6/CFP-10 and EspB/Rv3879c complexes. Both cytosolic complexes require interaction with Rv3871 for substrate secretion.

In this study, we identified EspB as a novel substrate of the ESX-1 secretion system and demonstrated a requirement for the *Mh3879c* and *Mh3871* genes in the secretion of EspB_M._ Further, we showed protein complex formation between EspB_M_ and Mh3879c, as well as identical behavior of their M. tuberculosis homologs. Two mutants of EspB_M_ that were stable after synthesis but failed to bind Mh3879c were not secreted, while a large carboxylterminal deletion did not interfere with either Mh3879c binding or secretion. Additionally, the carboxyterminus of Rv3879c/Mh3879c is required for interaction with and secretion of EspB. These results suggest that the EspB/Mh3879c protein complex is required for EspB_M_ secretion. While complex formation between ESAT-6 and CFP-10 is required for their secretion as a heterodimer by *M. tuberculosis,* Mh3879c appears not to be secreted. Our data, though, do not exclude the possibility that the aminoterminus of Mh3879c is quantitatively removed during or immediately after secretion, since we do not have and could not probe with antibodies to the native protein. We hypothesize that Mh3879c acts as a cytosolic chaperone to deliver EspB_M_ to the secretion machine. We showed that Rv3879c interacts directly with Rv3871 and that Rv3871, in addition to being required for the secretion of ESAT-6/CFP-10, is required for the secretion of EspB. Although our work does not reveal precisely how EspB is delivered to the ESX-1 machine, our data demonstrate that Rv3879c can interact with Rv3871 as well as with EspB_T_, suggesting that EspB may be targeted to Rv3871 in this way. We propose that the mechanisms of EspB and CFP-10 secretion intersect at binding to Rv3871 (Figure 7).

We also found that disruption of *Mh3868* leads to loss of accumulation of EspB in the bacterial cytosol. We previously observed that disruption of *Mh3868* prevents bacterial accumulation of ESAT-6 and CFP-10 [7]. *Mh3868* and its M. tuberculosis homolog *Rv3868* are predicted to be AAA ATPases, which suggests that they may function as chaperones for the translocation of ESX-1 substrates, but little is known about this key protein. We have found that *CFP-10* and *espB_M_* mRNAs are expressed in the *Mh3868::tn* mutants (B. McLaughlin and E. Brown, unpublished data), suggesting that the *Mh3868* gene product affects either the translation or stability of the ESX-1 substrates. Characterizing the function of Mh3868 will certainly be important to better understand ESX-1–mediated secretion.

Like ESAT-6 and CFP-10, EspA is secreted by the ESX-1 machine. Whether any of the M. marinum genes with sequence similarity to *espA* are functional orthologs has not yet been determined. Loss of either EspA or EspB inhibits secretion of ESAT-6 and CFP-10, but the reason for their requirement is unknown. It may be that as substrates reach the final common pathway for secretion, they interact in a manner that leads to cooperative secretion. Clearly, though, the secretion of EspB is quite distinct from that of EspA. While EspA requires CFP-10 for its secretion, EspB secretion is independent of CFP-10. EspB secretion is not disrupted in the M. marinum mutants *Mh3866::tn* and *Mh3867::tn,* neither of which secrete CFP-10, nor is EspB secretion disrupted in the *M. tuberculosis ΔCFP-10* mutant. These data are consistent with the model that EspB, unlike either ESAT-6 or EspA, is targeted to the ESX-1 machine independently of CFP-10.

These studies beg the question of whether it is possible to determine which ESX-1 substrates are most important for virulence. This has been a difficult task because of the apparent codependence of the various substrates on each other for secretion. However, our results allowed a somewhat different approach. We used a set of extRD1 mutants in which some *(Mh3866::tn* and *Mh3867::tn)* failed to secrete CFP-10, but did secrete EspB; while another mutant *(Mh3879::tn)* secreted CFP-10 but failed to secrete EspB; while mutants that disrupted the core secretion machinery *(MmΔRD1* and *Mh3871::tn)* and *espB_M_::tn* itself failed to secrete all substrates. We found that mutants lacking secretion of both substrates had a more marked growth defect in macrophages than the mutants lacking secretion of only one substrate. This suggests that the different substrates make distinct, and potentially additive, contributions to virulence. Although we cannot say that the defects in intracellular growth of the various mutants are caused by the substrates we have identified, our work does support the hypothesis that ESX-1 secretes more than one substrate that contributes to the virulence of *Mycobacteria* and that different substrates may have independent contributions to bacterial pathogenesis.

In summary, this work has identified a novel substrate for ESX-1–dependent secretion and has demonstrated interactions of this substrate with a protein encoded within RD1, expanding our understanding of how genes within this locus contribute to this novel secretion pathway. Furthermore, we have demonstrated that secretion of distinct ESX-1 substrates follows variable pathways to interaction with the core secretion machinery, and that the different substrates may contribute independently to intracellular survival and growth of the bacteria. These data extend the understanding of a major virulence mechanism of *Mycobacteria*.

## Materials and Methods

### Bacterial strains and plasmids.

All strains and plasmids used in this study are listed in Table 1. M. marinum strains were grown as previously described [24]. The designations assigned by the Sanger Institute in the annotation of the M. marinum genome and the corresponding DNA sequences are available at http://www.sanger.ac.uk/Projects/M_marinum/.

**Table 1:**
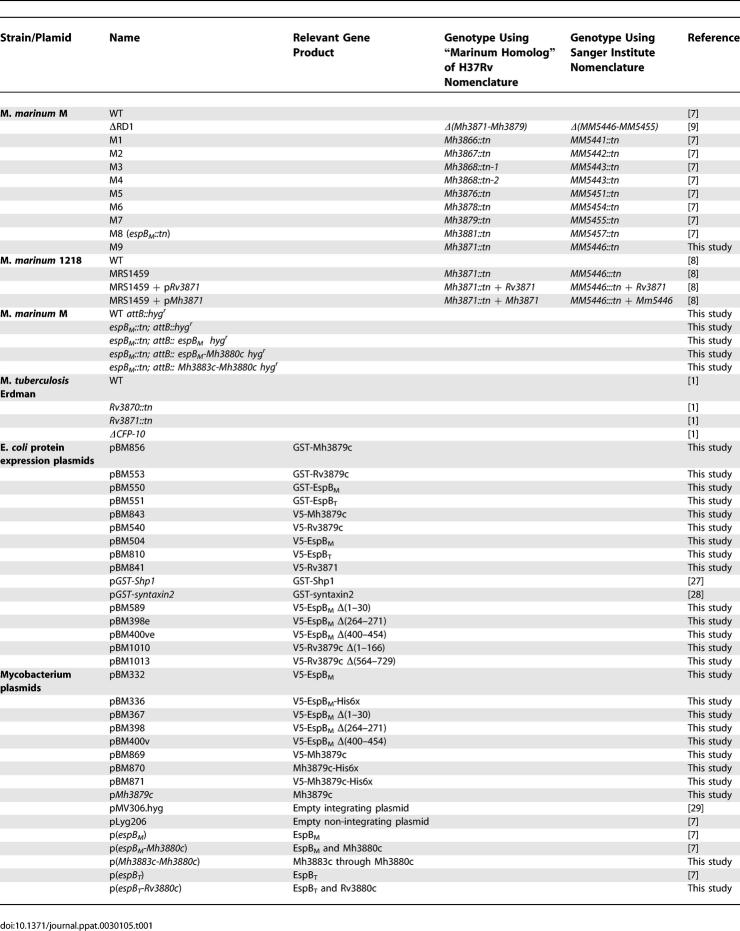
List of Bacterial Strains and Plasmids Used in This Study

The transposon insertion in the mutant *espB_M_::tn* lies between the 175th and 176th base pairs of the *espB_M_* gene, and the kanamycin gene within the transposon is transcribed opposite to the direction of transcription of the *espB_M_* gene. The strains M. marinum M *attB::hyg^r^* and *espB_M_::tn attB::hyg^r^* were constructed by transforming the strains M. marinum M WT and *espB_M_::tn* with the plasmid pMV306.hyg. The strains *espB_M_::tn attB:: espB_M_ hyg^r^* and *espB_M_::tn attB:: espB_M_ -Mh3880c hyg^r^* were constructed by ligating 250 bp upstream of *espB_M_* along with *espB_M_* or *espB_M_ -Mh3880c* into pMV306.hyg and then transforming the resulting plasmids, pBM264 and pBM262, into *espB_M_::tn.* The strain *espB_M_::tn attB:: Mh3883c-Mh3880c hyg^r^* was constructed by ligating 345 bp upstream of *Mh3883c* along with *Mh3883c-Mh3880c* into pMV306.hyg and then transforming the resulting plasmid, pBM263, into *espB_M_::tn.* To construct the plasmids pBM841, pBM540, and pBM810, the genes *Rv3871, Rv3879c,* and *Rv3881c* were PCR amplified from the cosmid RD1-2F9 [25] and ligated into pBM510, a derivative of pET22b+ in which the N-terminal His tag was replaced with the V5 epitope tag. To construct the plasmids pBM843 and pBM504, the genes *Mh3879c* and *Mh3881c* were PCR amplified from M. marinum M genomic DNA and ligated into pBM510. To construct the plasmids pBM332 and pBM336, a series of fragments were ligated into pLYG206 to achieve the following sequence ligated into the NotI and XbaI sites: 250 bp upstream of *espB_M_*, then the V5 epitope, then the *espB_M_* gene, and finally, in the case of the pBM336 plasmid, the His6x epitope. The plasmids pBM869, pBM870, and pBM871 were made in a manner synonymous to that of pBM332 and pBM336, where the *Mh3879* promoter and gene were used. The plasmids pBM367 and pBM400v were constructed by PCR from pBM332 and re-ligation of the truncated gene fragments back into pBM332, while pBM398 was generated by quick-change mutagenesis (Stratagene, http://www.stratagene.com/). For pBM589, pBM398e, and pBM400ve, the *espB_M_* gene fragments in the plasmids pBM367, pBM398, and pBM400v were cut by restriction digest and ligated into pBM504. For pBM856, pBM550, pBM553, and pBM551, the genes *Mh3879c, espB_M_*, *Rv3879c,* and *espB_T_* were cut by restriction digest from the plasmids pBM843, pBM504, pBM540, and pBM810 and ligated into the GST expression vector pGex-KG. To construct the plasmid p*Mh3879,* 250 bp upstream of the gene *Mh3879c* together with *Mh3879c* was PCR amplified from M. marinum genomic DNA and inserted into pLYG206. The plasmids pBM1010 and pBM1013 were constructed by restriction digests of pBM540 to excise portions of *Rv3879c,* and ligation of 5′ phosphorylated hybridized oligos that restored the frame and created the *Rv3879* deletions Δ(1–166) and Δ(564–729). To construct the plasmid p(*Mh3883c-Mh3880c*), the 345 bp upstream of *Mh3883c* along with *Mh3883c-Mh3880c* was cut by restriction digest from the plasmid pBM263 and inserted into pLYG206. To construct the plasmid p(*espB_T_-Rv3880c*), 250 bp upstream of the operon *Rv3881c-Rv3880c* together with the operon were inserted into pLYG206.

### Protein preparation and analysis.

M. marinum strains were grown in 40-mL cultures to 0.5 OD_600_ in 7H9 medium. The cultures were centrifuged and washed three times with 15 mL of PBS before re-suspension in 40 mL of Sauton's medium, supplemented with 0.015% Tween-80. When strains containing non-integrating plasmids for complementation were grown in Sauton's medium, the Sauton's medium was supplemented with Zeocin (5 μg/ml; Invitrogen, http://www.invitrogen.com/). After growth for 36 h at 30 °C, 105 rpm, in Sauton's medium, the cells were harvested by centrifugation. Supernatants were filtered through a 0.22-μm-pore-size filter with a glass pre-filter and concentrated with an Amicon Ultra-15 (5,000-molecular-weight cutoff; Millipore, http://www.millipore.com/) to 200 μL, which was saved as the culture filtrate (CF) fraction.

Pelleted cells were washed and resuspended in 1.5 mL of PBS with a protease inhibitor cocktail and 1 mM PMSF. Pellets were lysed using glass beads and the mini-bead beater (BioSpec Products, http://www.biospec.com/) with three 40-s pulses at maximum speed and incubations on ice in between each pulse, and then centrifuged at 3,000*g* for 2 min at 4 °C to remove unbroken cells. The resulting supernatant was collected and saved as the cell lysate (CL) fraction. M. tuberculosis (Erdman) culture filtrate and cell lysate fractions were prepared as previously described [1]. Total protein concentrations were determined by a Bradford assay.

### Western immunoblot assay.

Pellet and culture filtrate fractions were separated by SDS/PAGE on 10%–20% gradient polyacrylamide gels for detection of CFP-10; 7.5% polyacrylamide gels for detection of EspB, GroEL, or V5-tagged Mh3879; and 12.5% polyacrylamide gels for detection of FAP. Proteins were visualized by immunoblotting by using antibodies against EspB at a concentration of 1:500 (mouse polyclonal to the 100 amino acid fragment of Rv3881c [234–333 aa], Arizona State University CIM Antibody Core), and the blot was developed using ECL reagent West Dura (Pierce, http://www.piercenet.com/). Anti-CFP-10 (rabbit polyclonal; Colorado State University, http://www.cvmbs.colostate.edu/microbiology/tb/top.htm) was used at a concentration of 1:50000, blots of the culture filtrate fraction were developed using West Pico (Pierce), and blots of the cell lysates were developed using West Dura (Pierce). Anti-GroEL (rabbit polyclonal, SPA-875 / SPS-875; Stressgen, http://www.assaydesigns.com/) was used at a concentration of 1:10000, and blots were developed using West Pico (Pierce). Anti-FAP [22] for M. marinum samples was a rabbit polyclonal, used at a concentration of 1:10000 and developed using West Pico (Pierce). Anti-FAP for M. tuberculosis Erdman samples was CS-93 (Colorado State University), mouse monoclonal, used at a concentration of 1:20, and developed using West Pico (Pierce). His6x epitope was detected with a mouse monoclonal (Novagen, http://www.emdbiosciences.com/html/NVG/home.html) at a concentration of 1:1500, and V5 epitope was detected with a mouse monoclonal (R960–25, Invitrogen), at a concentration of 1:5000, and these blots were developed using West Pico (Pierce).

### Bacterial two-hybrid system assay.

The genes *Rv3614c, Rv3615c,* and *Rv3616c,* which were PCR amplified from genomic DNA, and each of the genes in the region *Rv3864* through *Rv3883,* which were PCR amplified from the cosmid RD1-2F9 [25], were cloned into the “bait” vector pBT (BacterioMatch II; Stratagene) in frame with cI. *Rv3881c* was cloned into the “target” vector pTRG in frame with the N-terminal subunit of RNA polymerase according to the manufacturer's instructions. The constructs were co-transformed into the E. coli two-hybrid system reporter validation strain XL1-Blue MRF′ *hisB lac* [*F*′ *laqIq HIS3 aadA Kanr*] and plated onto both the selective (+5 mM 3AT) and the non-selective screening medium according to the manufacturer's instructions. The non-selective screening plate is histidine-dropout M9 agar supplemented with 0.5 mM IPTG, 12.5 μg/ml tetracycline, and 25 μg/ml chloramphenicol. The selective screening plate is histidine-dropout M9 agar supplemented with 0.5 mM IPTG, 12.5 μg/ml tetracycline, 25 μg/ml chloramphenicol, and 5 mM 3-amino-1,2,4-triazole.

### GST pulldown.

GST fusion proteins, GST alone, and V5-tagged proteins were expressed in the BL21-RP codon plus E. coli strain (Stratagene) by addition of 0.2 mM IPTG (3 h at 30 °C). Bacterial cultures were lysed in buffer containing 50 mM HEPES (pH 7.4), 300 mM NaCl, 1% Triton X-100, 0.5 mM EDTA, and protease inhibitor cocktail (Roche). Solubilized proteins were separated by centrifugation at 20,000*g* for 10 min. The GST fusion proteins and GST alone were bound to glutathione agarose beads (Amersham Biosciences, http://www.gelifesciences.com) by incubation overnight at 4 °C. The beads were then extensively washed with PBS containing 0.1% Triton X-100. Bacterial lysates containing solubilized V5-tagged proteins, in lysis buffer, were incubated with the GST protein–loaded agarose beads overnight at 4 °C. After washing three times with PBS containing 1% Triton X-100, bead-bound protein was eluted in Laemmli buffer, seperated by SDS-PAGE, and analyzed by western blot.

### Macrophage infections.

All macrophages used in these experiments were derived from bone marrow cells of C57BL/6 mice that were differentiated for 6 d in DMEM supplemented with 10% CMG supernatant [26] and 10% fetal bovine serum (FBS; HyClone, http://www.hyclone.com/). Immediately prior to infection, macrophage monolayers were washed once with FBS-free DMEM. M. marinum strains were each grown to OD_600_ of 1.0, prepared for infection, and incubated with macrophages as previously described [7]. All infections were performed at a multiplicity of infection of 1, for 2 h at 32 °C, in a 5% CO2, humidified environment, in 24-well plates. The time at which M. marinum was added to the well was designated time zero. At the end of the 2 h incubation period (*T* = 2 h), infected monolayers were washed twice with DMEM and further incubated in DMEM containing 0.1% FBS and 200 μg of amikacin/ml for 2 h to kill extracellular bacteria. At the end of the antibiotic treatment, monolayers were washed twice with DMEM and incubated in DMEM containing 0.1% FBS at 32 °C and 5% CO2. Intracellular bacteria were enumerated by lysing macrophage monolayers and diluting and plating bacteria exactly as described [7]. Statistical analysis was performed by calculating the one-way analysis of variance (ANOVA) with GraphPad Prism 4.0 (GraphPad Software, http://www.graphpad.com).

## Supporting Information

Figure S1 Colony Morphology of *espB_M_::tn*
Dilutions of each strain were grown on 7H10 agar, without antibiotics, and imaged after 12 d.
(5.9 MB TIF)

Figure S2 *Mh3871* Is Required for the Secretion of EspB_M_
Cell lysates (CL) and culture filtrates (CF) were prepared from wild-type M. marinum strain 1218 and isogenic mutants as described in Materials and Methods. CL and CF proteins were separated by SDS-PAGE and the indicated proteins detected by western blot. Cultures of each strain were inoculated into Sauton's medium at an optical density (OD) of 0.5 and grown for 36 h. Therefore, the samples of each strain are normalized by OD readings. Of the total CL and CF fractions collected for each strain from one experiment, 3% of the CL was loaded in each lane and 15% of the total CF was loaded into each lane.
(1.0 MB TIF)

Figure S3 Mh3879c Is Not Secreted and the Carboxyterminus of Rv3879 Is Required for Interaction with EspB Cell lysates (CL) and culture filtrates (CF) were prepared from the indicated strains as described in Materials and Methods. Proteins were separated by SDS-PAGE and the indicated proteins were detected by western blot strains as described in Materials and Methods.
(A) Cultures of each strain were inoculated into Sauton's medium at an OD of 0.5 and grown for 36 h. Therefore, the samples of each strain are normalized by OD readings. Of the total CL and CF fractions collected for each strain from one experiment, 3% of the CL was loaded in each lane and 15% of the total CF was loaded into each lane.
(B) Agarose beads with immobilized GST only, GST-Rv3871, or GST-EspB_T_ were incubated with E. coli lysates that express V5-tagged Rv3879c full-length (1–729), Rv3879c Δ(1–166), or Rv3879c Δ(564–729). The input lysate (0.1%) and the material from the cell lysates that bound to the beads was run on SDS-PAGE and detected by western blotting with an antibody against V5.
(2.9 MB TIF)

### Accession Numbers

The GenBank (http://www.ncbi.nlm.nih.gov/Genbank/index.html) accession numbers for the gene products discussed in this paper are CFP-10 (NP_218391), ESAT-6 (YP_178023), Rv3866 (NP_218383), Rv3867 (NP_218384), Rv3868 (NP_218385), Rv3870 (NP_218387), Rv3871 (NP_218388), Rv3876 (NP_218393), Rv3877 (NP_218394), Rv3878 (NP_218395), Rv3879c (NP_218396), Rv3880c (NP_218397), and Rv3881c (NP_218398).

## Abbreviations

BMDM: bone marrow–derived macrophage
CFP-10: culture filtrate protein 10
EspB: ESX-1 substrate protein B
ESAT-6: early secretory antigenic target 6
ESX-1: ESAT-6 system 1
extRD1: extended region of difference 1
FAP: fibronectin attachment protein
OD: optical density
RD1: region of difference 1
